# Discrimination of Transgenic Maize Kernel Using NIR Hyperspectral Imaging and Multivariate Data Analysis

**DOI:** 10.3390/s17081894

**Published:** 2017-08-17

**Authors:** Xuping Feng, Yiying Zhao, Chu Zhang, Peng Cheng, Yong He

**Affiliations:** 1 College of Biosystems Engineering and Food Science, Zhejiang University, Hangzhou 310058, China; pimmmx@163.com (X.F.); zhaoyy@zju.edu.cn (Y.Z.); chuzh@zju.edu.cn (C.Z.); 2 Institute of Quality and Standard for Agro-Products, Zhejiang Academy of Agricultural Sciences, Hangzhou 310021, China; pc_phm@163.com

**Keywords:** classification, NIR hyperspectral imaging, chemometrics analysis

## Abstract

There are possible environmental risks related to gene flow from genetically engineered organisms. It is important to find accurate, fast, and inexpensive methods to detect and monitor the presence of genetically modified (GM) organisms in crops and derived crop products. In the present study, GM maize kernels containing both *cry1Ab/cry2Aj-G10evo* proteins and their non-GM parents were examined by using hyperspectral imaging in the near-infrared (NIR) range (874.41–1733.91 nm) combined with chemometric data analysis. The hypercubes data were analyzed by applying principal component analysis (PCA) for exploratory purposes, and support vector machine (SVM) and partial least squares discriminant analysis (PLS–DA) to build the discriminant models to class the GM maize kernels from their contrast. The results indicate that clear differences between GM and non-GM maize kernels can be easily visualized with a nondestructive determination method developed in this study, and excellent classification could be achieved, with calculation and prediction accuracy of almost 100%. This study also demonstrates that SVM and PLS–DA models can obtain good performance with 54 wavelengths, selected by the competitive adaptive reweighted sampling method (CARS), making the classification processing for online application more rapid. Finally, GM maize kernels were visually identified on the prediction maps by predicting the features of each pixel on individual hyperspectral images. It was concluded that hyperspectral imaging together with chemometric data analysis is a promising technique to identify GM maize kernels, since it overcomes some disadvantages of the traditional analytical methods, such as complex and monotonous sampling.

## 1. Introduction

Maize (*Zea mays* L.) is one of the most important agricultural commodities in the world, and also serves as a key ingredient in feed for livestock. It is used extensively in industrial products all over the world, including the production of renewable fuel [1]. The application of genetic transformation to maize has made rapid strides in the past decades to meet some specific requirements. Some agronomic traits, including enhancement of disease and insect pest tolerance [2], quality improvement [3], and increasing nutritional value [4], have been introduced into maize. In recent years, genetically-modified (GM) crop cultivation has been following the trend of combining two or more agronomical traits by transgenic breeding, referred to as “stacked” events [5]. The first binary transgenic event in GM maize production was mainly dominated by GM plants containing insect protection through endotoxin genes, conferred by *Bacillus thuringiensis* (*Bt*) as well as herbicide tolerance characteristics [5]. However, it has been argued that the use of GM techniques could possibly result in unpredictable adverse effects on food and environment safety. 

These unintended effects include the transfer of an uncontrollable escape of exogenous genes into neighboring wild plants by pollen, the formation of toxins associated with GM food, and modification of the biodiversity of the host plant by changing the expression of the existing genes [6]. The introduction of genetically-modified organisms (GMOs) in agro-food markets should be accompanied by a regulatory need to monitor and verify the presence and amount of GM varieties to guarantee consumer safety. Consequently, there is a need for GMO detection methods that are accurate, fast, and inexpensive. Currently, there are several analytical methods proposed for the determination, characterization, and authentication of GMOs in crops and derived crop products, such as polymerase chain reaction (PCR)/restriction enzyme assay [7], enzyme-linked immunosorbent assays [8], lateral flow strip [9], and microarray [10]. As a whole, the DNA- and protein-based methods for the identification of GMOs are versatile, sensitive, and accurate. However, there are also some disadvantages—they are destructive, laborious, expensive, time-consuming, and require highly-skilled operators; thus, they are unsuitable for online process control [11]. As non-destructive, synchronous, and coherent detection tools, spectroscopic techniques are environmentally friendly, fast, and easy to operate without complex sample pretreatments. 

The application of a method involving near-infrared (NIR) combined with chemometrics for the identification of GMOs in the agro-food market is feasible [12,13,14]. NIR is the region of the electromagnetic spectrum between 750 nm and 2500 nm, and NIR spectroscopy is often used to gather information on the relative proportions of C–H, N–H, and O–H bonds in organic molecules [11]. The basis of this technology for the detection of mutants, mediated by transgenic technology, is that it can identify phenotypic changes caused by genotypic changes, which ultimately bring about changes of organic molecular bonds [11]. Liu et al. (2014) distinguished GM rice seeds from their counterparts by using visible/near-infrared spectroscopy (VIS-NIR) spectroscopy combined with a chemometric tool with classification accuracy up to 100% with the least squares-support vector machine (LS-SVM) model [15]. Garcíamolina et al. (2016) applied NIR spectroscopy technology to discriminate GM wheat gain and flour from non-GM wheat lines [14]. Guo et al. (2014) also demonstrated that clear differences between GM and non-GM tomatoes could be identified by using VIS-NIR together with discriminant partial least squares regression with excellent classification accuracy of up to 100% [13]. However, conventional NIR—widely used for transgenic foods identification—lacks spatial dimension information. In contrast, NIR hyperspectral imaging combines traditional optical imaging and the spectral method which is capable of capturing images over broad contiguous wavelengths in the NIR region, and has received much attention in cereal science [16,17,18]. These images form a three-dimensional structure (x, y, λ) of multivariate data for processing and analysis, where x and y are the spatial dimensions (the number of rows and columns in pixels), and λ represents the number of wavelengths [19,20]. NIR hyperspectral imaging is a powerful spectroscopic tool for seed classification, quality discrimination, and detection of an object by obtaining visual information about the samples [21]. The benefits of using NIR hyperspectral imaging for cereal science are numerous, including disease and pest diagnoses [18,22], kernel density classification [16,17], seed moisture determination [23], and rice cultivar identification [24]. Currently, limited research has used this technique to distinguish GM from non-GM. Prior to this study, no research had mapped the spatial heterogeneity between GMOs from non-GM controls based on their different spectral signatures.

The purpose of this study was to investigate four goals: (1) to examine the feasibility of using NIR hyperspectral imaging techniques to identify GM maize kernels mediated by *Agrobacterium tumefaciens* and detect spatial heterogeneity in spectral variability; (2) to identify important wavelengths that identify the differences between GM and non-GM maize kernels; (3) to build an optimal discrimination model based on these important wavelengths to simplify the prediction model and to speed up the operation; and (4) to visualize the number and locations of GM maize kernel by developing imaging processing algorithms.

## 2. Materials and Methods 

### 2.1. Maize Samples

**Figure 1:**
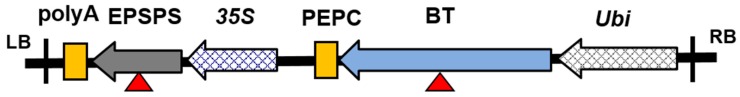
Structure of the plant expression vector containing coding regions of the *cry1Ab/cry2Aj-G10evo* genes. LB is left border; RB is the right border; poly A is a terminator; PEPC is a terminator; 35S is a promoter; Ubi is a promoter; EPSPS denotes the herbicide-resistant genes; BT denotes the insect-resistant genes; EPSPS and BT are marked with a red triangle.

The GM maize kernels used in this study (containing insecticidal and herbicide tolerant traits, *cry1Ab/cry2Aj-G10evo* genes) and their non-GM control were provided by the Institute of Insect Sciences, Zhejiang University, China. For the test maize, variety zhengdan958 was used as the GM acceptor line. Glyphosate tolerance of maize was obtained by expression of a mutant 5-enolpyruvylshikimate-3-phosphate synthase (EPSPS) enzyme. Insect resistance of the maize was obtained by expression of a *Bacillus thuringiensis* delta endotoxin protein. The transgenic maize was created by an *Agrobacterium tumefaciens*-mediated transformation system (Figure 1). There were no other differences between the transgenic maize and the non-transgenic control kernels. The GM and non-GM maize crops were grown in the same field to eliminate any environmental effects.

Intact samples of 1050 transgenic maize kernels and 1050 non-transgenic maize kernels were used for image acquisition. In total, 1050 samples of each genotype were randomly selected to form the calibration and prediction sets in a ratio of 2:1. Thus, there were 700 samples used for the calibration set and 350 samples used for the prediction set. Samples were classified according to the genetic background by classification model, which preferably should be approximate to the values assigned. In this study, the spectral data from GM maize kernels were assigned 1, and those of non-GM maize kernels were assigned 2.

### 2.2. Near-Infrared Hyperspectral Imaging

A ground hyperspectral imaging system was used to acquire NIR hyperspectral images. This system’s equipment mainly consists of the following devices: a N17E-QE imaging spectrograph (Spectral Imaging Ltd., Oulu, Finland), two 150 W tungsten halogen lamps (Fiber-Lite DC950 Illuminator; Dolan Jenner Industries Inc., Boxborough, MA, USA) for illumination, a high-performance CCD camera (Hamamatsu, Hamamatsu City, Japan) coupled with a C-mount imaging lens (OLES22; Specim, Spectral Imaging Ltd., Oulu, Finland), a displacement platform driven by a stepper motor (Isuzu Optics Corp., Zhubei, Taiwan) to move the samples, and a computer. The hyperspectral imaging system acquires spectra in the form of pixels from the range of 874–1734 nm with a spectral resolution of 5 nm intervals. Maize kernel samples were positioned on the conveyer belt. The exposure time was set to 3 milliseconds, and the distance between the lens of the CCD camera and the sample was set to 258 mm. Maize kernels were placed on the conveyor stage and moved with a speed of 19 mm/s to be scanned. 

Before spectral data and image processing, the acquired raw images must be corrected, and the calibrated image *R* was calculated using the following equation:

(1) $$R=\frac{I_{raw}-I_{dark}}{I_{white}-I_{dark}}$$

where *I_raw_* is the raw hyperspectral image; *R* is the calibrated hyperspectral image; *I_dark_* is the dark reference image by turning off the light source with reflectance close to 0; and *I_white_* is the white reference image by using a white Teflon tile with 100% reflectance.

### 2.3. Spectral Collection and Pretreatment

To extract spectral data, the whole maize kernel was segmented from the background and the region of interest (ROI) was defined. The spectral mean of all the pixels of the ROI was taken as the average spectrum of the relative sample. For the purpose of eliminating the noise of the spectral data and to improve the predictive ability of the samples, three typical pre-processing methods were used—namely, wavelet transformation (WT) [25], standard normal variate (SNV) [26], and multiplicative scatter correction (MSC) [26]. The raw spectra were subjected to noise suppression by wavelet transformation using Daubechies 8 with decomposition scale 3, which was conducted by a series of MATLAB programs. SNV and MSC pre-processing was implemented using the Unscrambler software version 10.1 (CAMO PROCESS AS, Oslo, Norway). 

### 2.4. Multivariate Chemometrics Analysis

**Figure 2:**
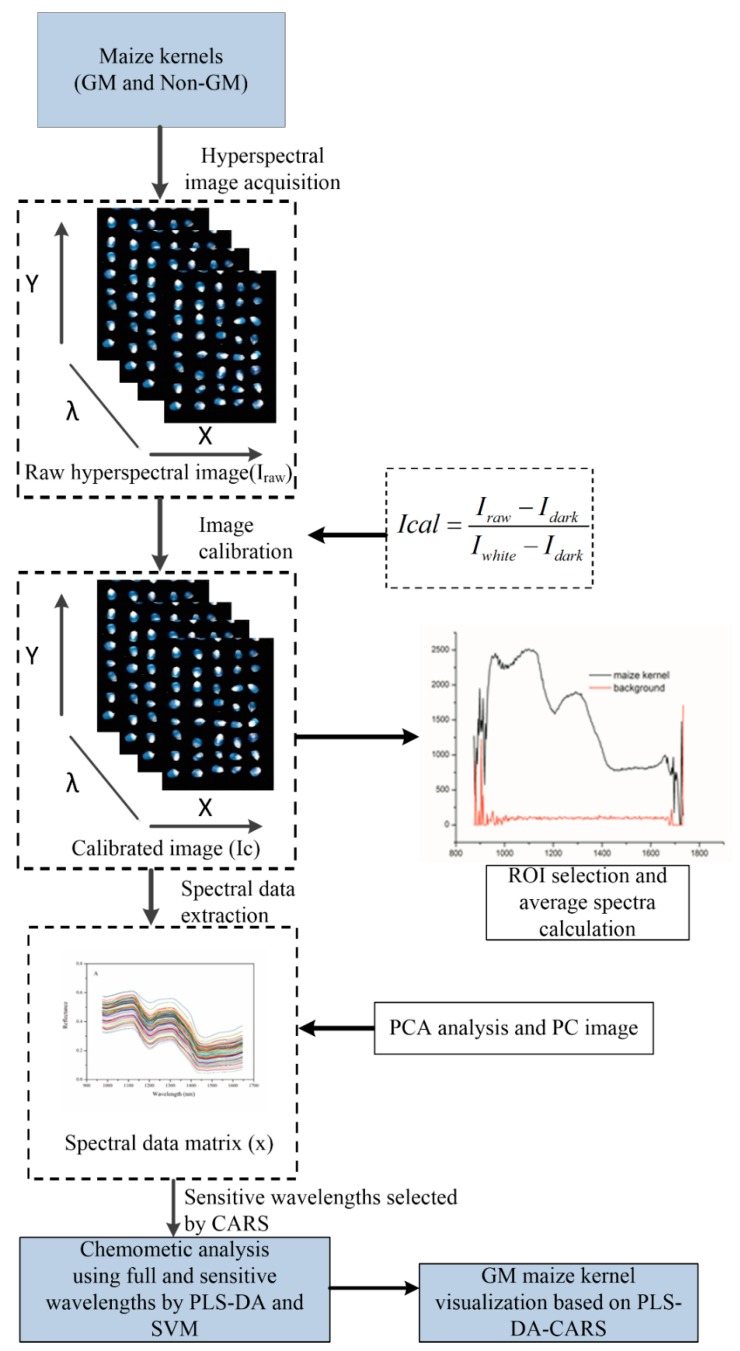
Flowchart of image processing and data analysis for discrimination of genetically-modified (GM) maize kernels. CARS: competitive adaptive reweighted sampling; PLS-DA: partial least squares discriminant analysis; ROI: region of interest; SVM: support vector machine.

Multivariate analyses including principal component analysis (PCA), partial least squares discriminant analysis (PLS-DA), and support vector machine (SVM) were used in the present study to classify and screen the GM and non-GM maize kernels. Exploratory classification was carried out by PCA analysis in order to find possible clustering by their average spectral characters. The contiguous spectral bands in hyperspectral image data are highly correlated, and thus the high dimensionality results are redundant information. It is essential to extract feature components to augment both efficiency and effectiveness. Next, competitive adaptive reweighted sampling (CARS) [27] was applied to select the important wavelengths. In the next stage, the PLS-DA and SVM discriminant analysis models were established based on the raw average spectral datasets (200 wavelengths) and optimal spectra (54 wavelengths) of all test samples. Finally, a prediction map was developed by applying the CARS-PLS-DA model based on each pixel at the optimal wavelengths. Image visualization helped to present the distribution of different features between different genotypes. In general, the prediction map is presented in a pseudo-color map, and the colors represent the corresponding feature values. The hyperspectral image processing procedure is illustrated in Figure 2, and includes spectral data extraction, optimal wavelengths selection, the development of discrimination models, and the building of a prediction map. 

PCA is an effective algorithm for reducing the dimensionality of data into a set of principal components (PCs), for solving the problem of multicollinearity and handling any potential co-linearity between variables [28]. The PCA algorithm transforms multiple variables into a smaller number of PCs. First, exploratory classification was carried out by PCA analysis to identify clusters into the genetic background classes—GM and non-GM—based on their average spectral data. Because the PCs are orthogonal, we can view the possible distinction between different samples by plotting the PCs. PCA score images of the first three scores were conducted by combing all-pixel spectral information and then the score information in the next step. Anomalies in the interpretation of PCA score images between different genotypes would most likely be due to chemical components in heterogeneity [29]. According to Wold et al. (1996), discriminant analysis models established on optimal wavelengths might have the same or better results than those established with full spectra [30]. Moreover, the reduced number of wavelengths makes the model easier to apply and is sufficient to determine if classification works [31]. CARS is a promising procedure for variable selection and was applied in this work. The number of Monte Carlo sampling runs was set to 50, and 10-fold cross validation was used to evaluate the effectiveness of each subset of variables. The CARS method was implemented in MATLAB with open script code which is available at http://cn.mathworks.com/matlabcentral/fileexchange/64154-cars-algorithm-for-feature-variable-selecting. A detailed description of the CARS procedure can be found in Li et al. (2009) [27]. 

PLS-DA is a supervised method used for classification purposes to explain the maximum discrimination between defined samples groups [32]. PLS-DA linearly models the relevant sources of data into new variables called latent variables (LVs), and the first few LVs carry the most useful information. In this case, the PLS-DA discrimination model was built by assigning reference values for all the samples. The GM maize kernel would be considered to be correctly evaluated if the value was between 0.5 and 1.5. A sample was considered non-GM if the value was between 1.5 and 2.5. Otherwise, the samples were considered as incorrectly classified. The PLS-DA model was built using leave-one-out cross validation, and the number of optimal LVs was determined. The accuracy of the classification procedure is expressed as the fraction of correctly classified samples to the total samples for both the calibration and prediction sets.

SVM is a supervised learning model based on structured risk minimization that analyzes data used to perform multivariate function estimation or a non-probabilistic binary linear classification [33]. Compared to other machine learning methods, this method develops a model with less training samples, and overcomes the local minimum required for a neural network. SVM has been widely used for supervised pattern recognition. Detailed information about this popular model can be found in the literature [34]. For this study, SVM with the radial basis function (RBF) as the kernel function was used, and different penalty parameters (c) and kernel function parameters (g) were chosen to achieve the highest recognition rate. The best c and g were obtained by a grid-search procedure in the range of 2^−8^–2^8^ with the kernel function of RBF.

### 2.5. Software Tools

Images were analyzed by using Evince version 4.6 Hyperspectral image analysis soft package (ITT, Visual Information Solutions, Boulder, CO, USA) and MATLAB version R2010b (The Math-Works, Natick, MA, USA). In addition, origin Pro 7.0SR0 (Origin Lab Corporation, Northampton, MA, USA) software was used to design graphs. The model performance was evaluated by the classification accuracy of the calibration set and the prediction set. 

## 3. Results and Discussion

### 3.1. Spectroscopic Analysis

**Figure 3:**
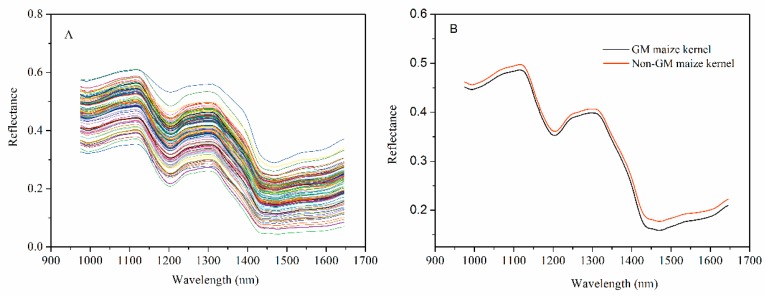
The profiles for raw spectra (**A**) and average spectra reflectance (**B**) from the near-infrared (NIR) multispectral images of GM and non-GM maize kernels.

The spectra were collected over the range of 874–1733 nm. Only the spectra of 971.66–1642.43 nm were used for analyses, as the front and rear parts of the spectra showed high noise levels caused by the optical equipment and the ambient environment. Figure 3A shows the extracted spectra of the ROI, and Figure 3B represents the average spectra of 1050 transgenic and 1050 non-transgenic maize kernel samples. The differences in spectra reflectance were observed, noting that the trends of most spectra were similar. The average reflectance of the non-GM samples was always higher than those of the GM samples, which reflects the differences in the hundreds of physical and chemical components between the genotypes. These differences might result from metabolites in the transgenic samples. It was hard to discriminate GM samples from their non-GM control based on the NIR spectral reflectance only. Therefore, chemometrics methods in combination with NIR spectra were introduced to build the discriminant analysis models for classification.

### 3.2. Spectral Analysis by Principal Component Analysis

Spectra data were pre-processed to eliminated the systematic noise and highlight the differences between the samples. PLS-DA was applied using leave-one-out cross-validation for the original raw spectral data and the pre-processed spectra to test the different pre-treatment strategies. Table 1 summarizes the results acquired for raw spectra and the different pre-processing methods. In all cases, the optimal number of LVs for establishing the calibration set was nine. Discrimination performance of the calibrations can be improved by each pre-processing treatment, but the performance of the prediction model was only improved by WT pre-processing. From the different pre-treatments evaluated, WT correction was the most efficient pre-treatment. In order to establish a robust prediction model, WT was applied as the pre-treatment method in the next step.

**Figure 4:**
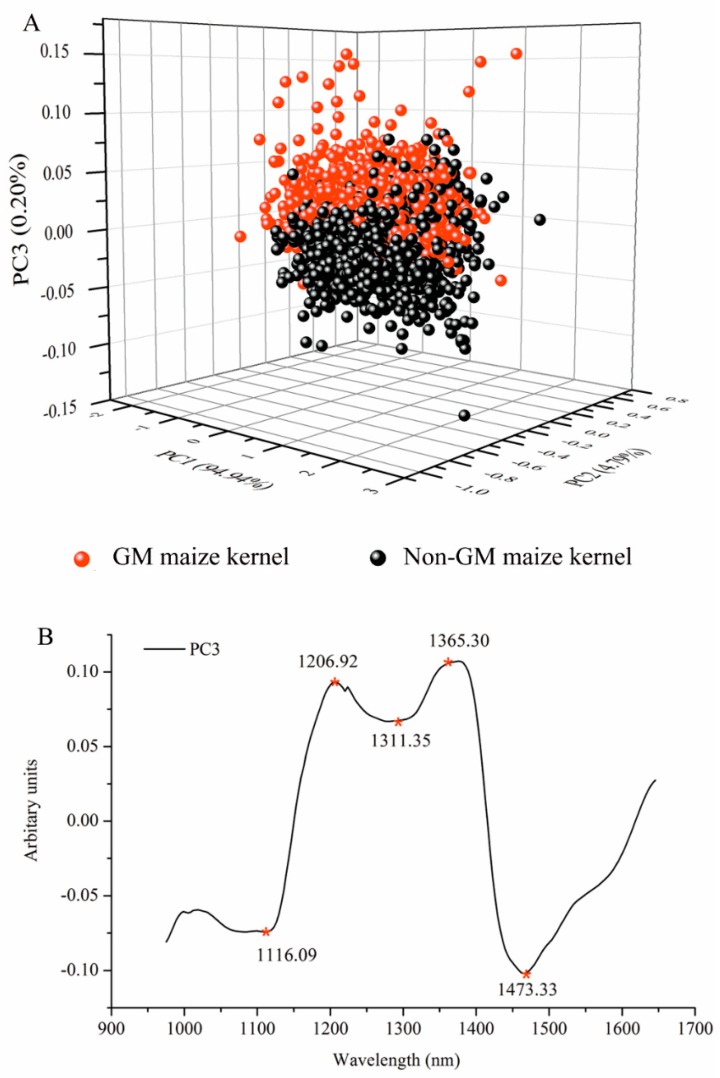
Three-dimensional principal component analysis (PCA) scores scatter plot of the first three principle components (PCs) for the GM and non-GM maize kernels with raw spectra (**A**) after pre-processing and (**B**) main peaks of PC3 loadings that are indicative of the differences between GM and non-GM maize kernels.

After WT was applied, PCA programs were first developed to examine the qualitative difference of GM and non-GM maize kernels in PC space. All spectra of the 1050 GM and non-GM maize kernels were analyzed for PCA. The three-dimensional (3D) PC score plot of the samples is illustrated in Figure 4A. The first three PCs explained the most spectral variations, at a total of 99.02%, including 94.04%, 4.79%, and 0.20% for PC1, PC2, and PC3, respectively. It was evident that the two classes were well-separated along the third PC, which indicated that the spectral fingerprints carry discriminant information. The suitability of PCA for distinguishing *Bacillus thuringiensis*-mediated transgenic rice seeds from NIR has been previously demonstrated [15]. 

**Figure 5:**
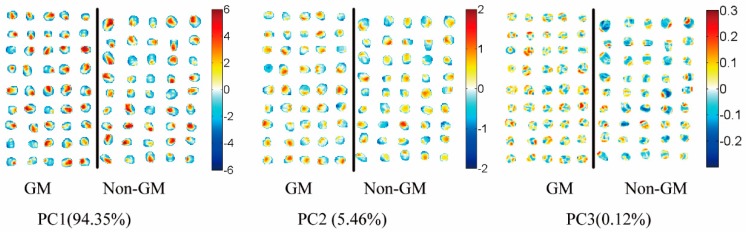
Score images of the first three principal components (**PC1**–**PC3**) of the images of maize kernels.

Since hyperspectral imaging possesses all-pixel spectral information, PCA visualization analysis on hyperspectral reflectance images was also introduced, instead of using the average spectrum of each sample. Score images (Figure 5) were investigated to identify and visualize the patterns detected on the score plots. The score plot of PC1 and PC2 did not show clear classification differences between genotypes, as these PCs were associated with maize kernel composition and anatomy [16,17]. The introduced foreign genes did not change the anatomical properties of the kernel and major kernel dominant traits, such as protein, fat, and starch concentration. Maize kernel mainly consists of two types of endosperm texture. In the vitreous endosperm, starch granules are polygonal-shaped and tightly compacted without air spaces. The floury endosperm comprises spherical starch granules that are covered with a protein matrix and air spaces [29]. The main source of spectral variation was explained by PC1. The germ region and pedicle of the maize kernel is composed of a floury endosperm, while the other pericarp of the kernel is composed of a glass endosperm [35]. As illustrated in Figure 5, the positive PC1 scores (shown in red color) were associated with floury endosperm in the germ and pedicle region, while negative PC1 scores (shown in blue color) were associated with the glass endosperm. The score image of PC2 showed different features linked to the pedicle and hull of maize’s histological characteristics, as earlier described by Williams (2016) [17]. Similar findings regarding morphological classes including vitreous and soft endosperm were also reported by other researchers [16,17,28]. With the score image of PC3, the first visualization of a difference between GM and non-GM maize kernels was observed (Figure 5). The GM samples were largely characterized by positive scores (shown by the colors in the warm range) on the surface of the kernels, while non-GM samples were mainly covered by cool colors. The differences observed were the same as in the PCA 3D plot using the spectral data.

The value of the PCA loadings reflects the degree of correlation between the PCs and the raw wavelength variable; therefore, the variation observed in the PCA score plots and images can be explained by studying the accompanying loading. The variation is explained by the loading line plot of PC3 (Figure 4B). The absorption bands around 1206 nm are related to the second overtone of C–H stretching vibration of various functional groups: –CH_2_, –CH_3_, and –CH=CH– [36]. The peak near 1311 nm is due to the first overtone of the OH stretch and OCO bending [37]. The remarkable peak centered around 1365 nm is related to the C–H_3_ stretch and deformation overtone [38]. The band around at 1473 nm represents OH, CH, and CH_2_ deformations [39]. 

### 3.3. Selection of Optimal Wavelengths

**Figure 6:**
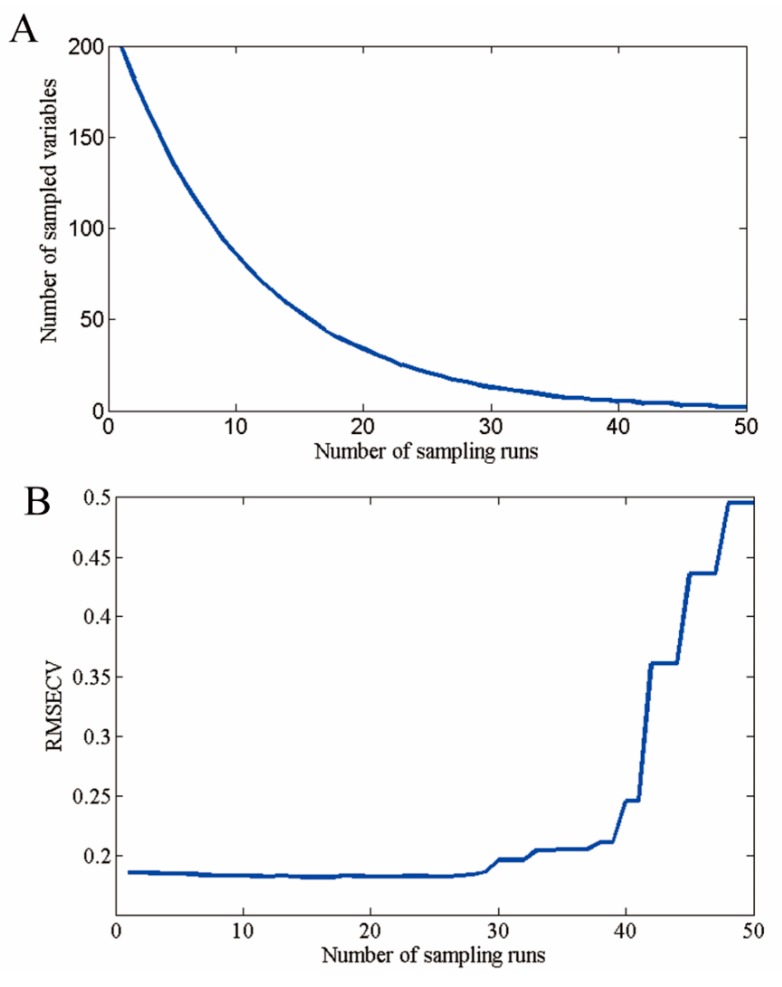

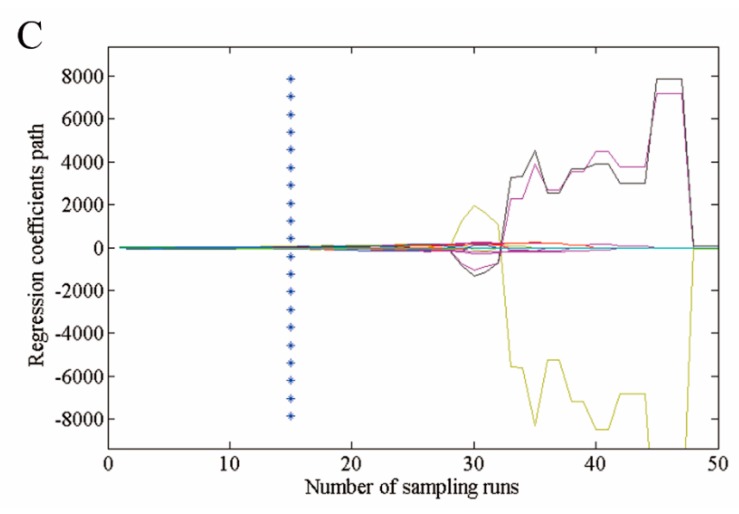
Selected optimal wavelengths by competitive adaptive reweighted sampling (CARS). (**A**) The number of variables as the function of iterations; (**B**) ten-fold root mean squared errors (RMSECV) values; and (**C**) regression coefficients of each variable with the number of sampling runs. Each line denotes the path of a variable.

**Figure 7:**
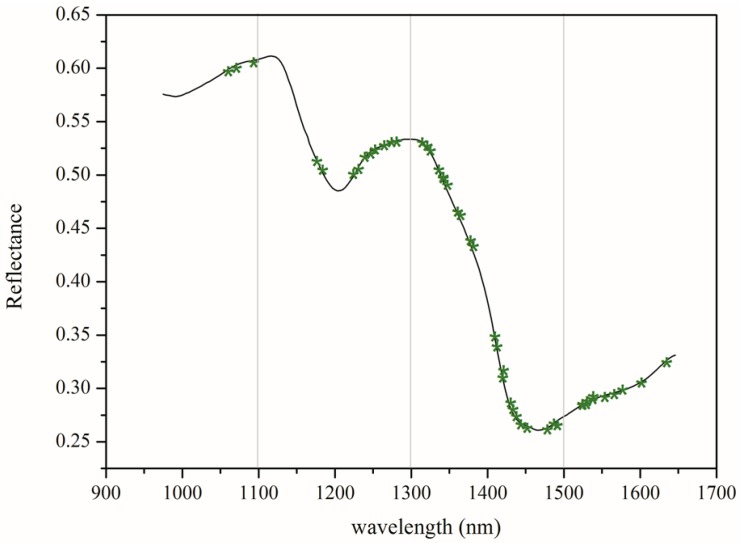
The distribution of optimal wavelengths selected by CARS.

Hyperspectral imaging data contain redundant information, which affects the prediction performance of the model. Variable selection was carried out using CARS election-based techniques to reduce the effect of non-related variables and speed up the classification. As shown in Figure 6 and Figure 7, 54 optimal wavelengths were selected. The wavelength number was decreased by 73% ($\frac{200-54}{200}$ = 94%) after preprocessing all the wavelengths by CARS. 

The bands found between 1250–1350 nm were due to the combination between the first overtone of Amide B with the fundamental Amide III vibrations [40]. The spectral region (1410–1480 nm) was assigned for protein as a result of the first overtone of the N–H stretching vibration [40]. The bands at 1520–1600 nm were related to the N–H stretching vibrations [41]. Based on the above interpretations and observations, it is reasonable to assume that the change in conformation and composition status of the GM maize is due to the pleiotropic effect caused by the insertion of *cry1Ab/cry2Aj-G10evo* foreign genes into the parent genome, influencing the NIR spectra and causing variation between the different genotype backgrounds.

### 3.4. Classification Analysis by the Discrimination Model 

In the next stage, spectra collected from the images of the kernel samples were used to build a model capable of discriminating the GM maize kernels based on their hyperspectral fingerprint. Calibration model measurements were conducted on the full spectrum of 200 wavelengths, and 54 optimum wavelengths were selected. The recognition effect of different discrimination models, developed with full and selected feature wavelengths, are compared in Table 2. The recognition accuracies obtained from the calibration and prediction sets were summarized. The SVM and PLS-DA models all achieved good recognition results with large sample size. The classification ability of the PLS-DA model was higher than that of SVM when all spectrum regions were used. The calibration set was 99.43% accurate for PLS-DA and 98.5% for SVM. The prediction set was 98.71% accurate for PLS-DA and 97% for SVM. The CARS algorithm was used to select optimal wavelengths from NIR hyperspectral imaging. The number of effective wavelengths decreased to 27% after using this algorithm. The variable selection made the modeling procedure faster. The discrimination ability of the calibration set from the PLS-DA model, based on optical wavelengths, was slightly worse than that obtained from all the wavelengths, but was still rated as acceptable. The discrimination ability of the prediction set from the PLS-DA model increased from 98.71% to 99.00%. The SVM model established on selected wavelengths performance improved, since it was 99.14% accurate with the calibration set and 98.29% for the prediction set. The reason for this might be that some wavelengths carrying useless interference were eliminated. Comparison of the results showed that CARS-PLS-DA performs better than CARS-SVM, since it made the prediction more robust and accurate. The overall results indicated that it was feasible to discriminate GM maize kernel by using hyperspectral imaging, and that the PLS-DA recognition model based on optimal wavelengths is a reliable and robust model. 

### 3.5. Transgenic Maize Kernel Visualization

**Figure 8:**
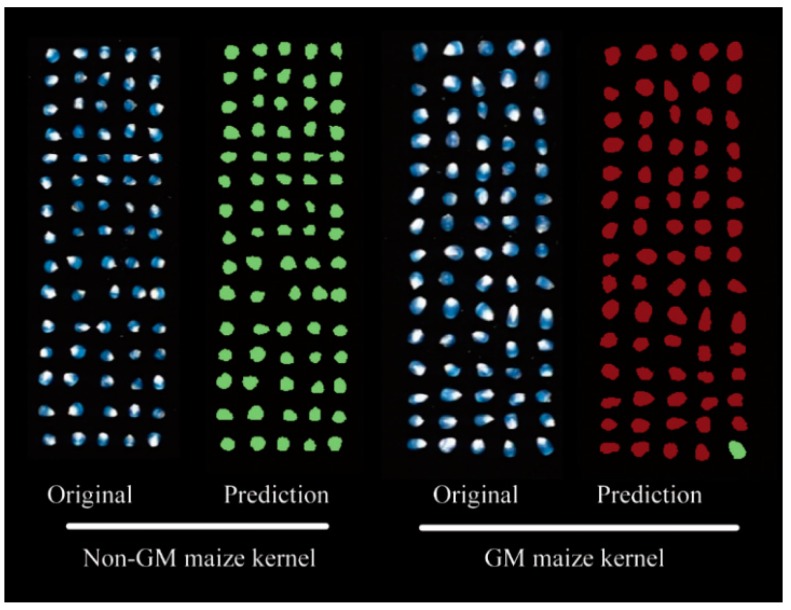
Visualization of independent maize kernels by the SVM classification process using optimal wavelengths in the hyperspectral images. Green denotes non-GM maize kernels, and red identifies GM maize kernels.

In addition to verifying the reliability of the proposed method, the classification of genotypes was visualized on prediction maps by predicting the features of each pixel on individual hyperspectral images. Accordingly, the PLS-DA model—computed using optimal wavelengths selected by CARS—was applied to every single pixel in the image to predict the class of kernels (GM and non-GM maize) for all surfaces of the sample. For creating a classification map, a binary code with a dummy variable was used to classify samples, with GM samples assigned as one and non-GM samples assigned as two. The result is shown in Figure 8 with pixels in prediction map colored according to the predicted category with the same dimension as the original hyperspectral image. Although it was difficult to determine the difference between the two classes from sample to sample and from point to point with the naked eye (Figure 8), GM maize kernels were obviously identified from the final chemical image. Green represents the non-GM maize kernel, and the red the GM maize kernel. Notably, some kernels on the classification map were misidentified based on the CARS-PLA-DA model. The morphological characteristics of the kernels in the classification map were altered due to the low resolution of the NIR imaging system and the image segmentation algorithm. However, the main shape of the kernels and their locations were clear on the prediction map. This approach is important because it facilitates the progress for rapid and high throughput detection of GM maize kernels and could be implemented as an online visualization system for iscrimination purposes.

## 4. Conclusions

The above results demonstrate that it is possible to differentiate a stacked commercial maize hybrid containing both herbicide-tolerant and insect-resistant traits from a single transgenic event by coupling the hyperspectral imaging technique in the NIR region (1975.01–1645.82 nm) with chemometric processing. Both PCA and classification models were suitable for GM maize kernel variety identification. From the perspective of the pixel spectra combined with the spatial distribution of the maize kernel, a principal component pseudo-color map was drawn and the differences were intuitively displayed. High-dimensional hyperspectral image data were reduced by CARS to extract the characteristic spectrum. The classification models built by PLS-DA and SVM using full wavelengths had a predictive accuracy near 100%. Additionally, it was demonstrated that the PLS-DA model established with a reduced set of only 54 wavelengths resulted in excellent accuracy, with 99.35% for the calibration set and 99.00% for the prediction set. This last outcome was fairly promising, as it could significantly speed up the data processing, which could facilitate online detection in the future. The main benefit of the hyperspectral imaging technique is its ability to visualize the identification of GM maize kernel in a pixel-based manner that cannot be obtained with either common spectroscopy or imaging. Finally, the GM maize kernel could be identified on the prediction maps by using the features of each pixel on individual hyperspectral images obtained by the CARS-PLS-DA model. We conclude that it is feasible to use hyperspectral imaging to differentiate GM maize kernels from their non-GM parents. The experiment material used for classification were obtained from the same year. Further research is expected to use the seeds or kernel samples from different years, regions, and transgenic events involved to improve the reliability and adaptability of the discrimination model.

## Figures and Tables

**Table 1:** Comparison of discrimination performance obtained by partial least squares discriminant analysis (PLS-DA) with different preprocessing methods using full wavelengths.

Methods	PLS-DA		
	Parameter ^1^	Calibration Set	Prediction Set
**Raw**	9	98.50%	97.86%
**WT**	9	99.43%	98.71%
**SNV**	9	99.50%	95.00%
**MSC**	9	99.36%	94.57%

^1^ Model parameters means the optimal number of LVs for establishing the calibration model of PLS-DA; Raw means raw spectra; WT is wavelet transformation using Daubechies 8 with decomposition scale 3; SNV is standard normal variate; and MSC is multiplicative scatter correction.

**Table 2:** Comparison of discrimination ability obtained by different PLS-DA and SVM models, with the total spectral data and optimal wavelengths selected by the competitive adaptive reweighted sampling (CARS) method.

Methods	PLS-DA			SVM		
	Parameter ^1^	Calibration Set	Prediction Set	Parameter ^1^	Calibration Set	Prediction Set
**Full spectra**	9	99.43%	98.71%	(256, 0.0625)	98.5%	97.00%
**Optimal wavelengths**	8	99.35%	99.00%	(256, 16)	99.14%	98.29%

^1^ Model parameters of the differentiating models; i.e., the optimal number of LVs for establishing the calibration model of partial least squares-discrimination analysis (PLS-DA), different penalty parameters (c) and kernel function parameters (g) for support vector machine (SVM).
